# Recent Advances in the Bacterial Phytohormone Modulation of Plant Growth

**DOI:** 10.3390/plants12030606

**Published:** 2023-01-30

**Authors:** Ma. del Carmen Orozco-Mosqueda, Gustavo Santoyo, Bernard R. Glick

**Affiliations:** 1 Departamento de Ingeniería Bioquímica y Ambiental, Tecnológico Nacional de México/I.T. Celaya, Celaya 38110, Guanajuato, Mexico; 2 Genomic Diversity Laboratory, Institute of Biological and Chemical Research, Universidad Michoacana de San Nicolás de Hidalgo, Morelia 58030, Michoacan, Mexico; 3 Department of Biology, University of Waterloo, Waterloo, ON N2L 3G1, Canada

**Keywords:** cytokinin, gibberellin, volatile organic compound, salicylic acid, auxin, ethylene

## Abstract

Phytohormones are regulators of plant growth and development, which under different types of stress can play a fundamental role in a plant’s adaptation and survival. Some of these phytohormones such as cytokinin, gibberellin, salicylic acid, auxin, and ethylene are also produced by plant growth-promoting bacteria (PGPB). In addition, numerous volatile organic compounds are released by PGPB and, like bacterial phytohormones, modulate plant physiology and genetics. In the present work we review the basic functions of these bacterial phytohormones during their interaction with different plant species. Moreover, we discuss the most recent advances of the beneficial effects on plant growth of the phytohormones produced by PGPB. Finally, we review some aspects of the cross-link between phytohormone production and other plant growth promotion (PGP) mechanisms. This work highlights the most recent advances in the essential functions performed by bacterial phytohormones and their potential application in agricultural production.

## 1. Introduction

**Figure 1:**
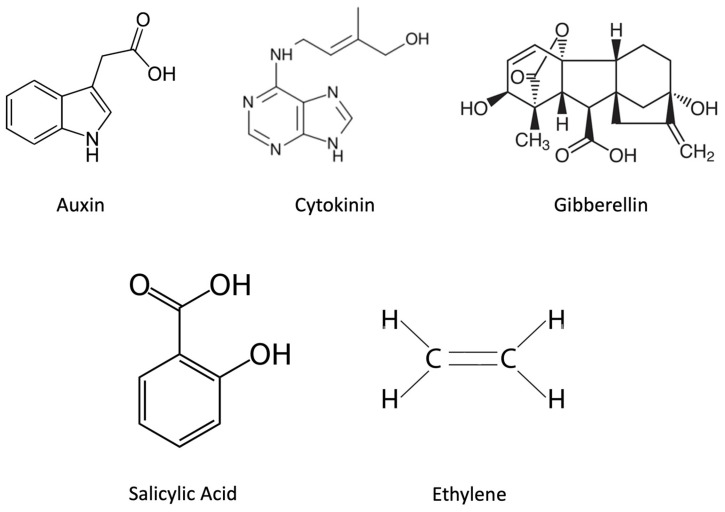
Chemical structures of PGPB-synthesized phytohormones.

Plant physiological activities are regulated by the action of several different phytohormones including cytokinin, gibberellin, abscisic acid, salicylic acid, jasmonic acid, brassinosteroids, auxin, and ethylene. Interestingly, many plant growth-promoting bacteria (PGPB) can also synthesize or degrade some of these phytohormones including cytokinin, gibberellin, salicylic acid, auxin, and ethylene (Figure 1) [1,2,3,4,5]. In addition, many PGPB synthesize a range of different volatile organic compounds (VOCs) [6,7]. Some of these VOCs can directly promote plant growth while others function by modifying the expression of the abovementioned phytohormones. Both plant- and PGPB-encoded phytohormones modulate plant growth and development and help plants to respond to a variety of environmental changes (both biotic and abiotic). Various phytohormones function by either activating or deactivating the expression of other plant genes that help the plant in responding to a range of environmental challenges. Moreover, to maintain maximally effective levels of various phytohormones, plants may utilize the phytohormone-degrading activity that exists in many soil bacteria [5,8].

The basis of much of the current knowledge of the detailed mode of action of various phytohormones comes from adding purified hormones to plants under laboratory conditions and documenting the effect of these hormones on the plants’ growth and development. However, it is difficult to know the precise amount of an added phytohormone (added either as a purified chemical or as a phytohormone produced by a PGPB) that has been incorporated into a plant and its target tissue(s). In addition, the concentration and activity of a particular phytohormone may affect the synthesis or degradation of other phytohormones within a plant’s tissues. Moreover, the majority of PGPB can synthesize, or modulate the concentrations of, several phytohormones so that it is often not possible to ascribe a plant physiological response to a PGPB to one specific phytohormone. To understand the functioning of a particular PGPB-produced phytohormone in promoting plant growth, it is best to create phytohormone minus or overproducing mutants of that PGPB and study the effects of the mutant PGPB strains compared to the wild-type strain on plant growth and development. Due to the importance of understanding the modulation of phytohormones by beneficial bacteria and the rapid progress of this field, in this work we review the most recent advances in these regulatory molecules of plant growth and development, including those areas that are related to sustainable agriculture.

## 2. Cytokinin

The structure of cytokinins (CKs) is similar to the structure of adenine. The most common form of cytokinin in plants is zeatin which was first isolated from *Zea mays* (corn) in the 1950s [9]. Cytokinins can promote plant cell division or cytokinesis [10]. In addition to plant-synthesized cytokinins, several bacteria (both PGPB and phytopathogens) can synthesize cytokinins including zeatin, zeatin riboside and isopentenyladenine. Based on both biochemical and DNA sequence data [11], only a limited number of soil bacteria synthesize cytokinin and it appears that phytopathogens synthesize a much higher level of cytokinins than PGPB.

Cytokinins have an effect on many different types of plant cells. They can modulate cell division, seed germination, apical dominance, root elongation, xylem and chloroplast differentiation, transition to the reproductive growth phase, flower and fruit development, leaf senescence, nutritional signaling, and plant–pathogen interactions [12,13,14]. Since cytokinins promote plant cell division, they consequently inhibit plant senescence. In plants growing in nature, a higher ratio of cytokinins to auxins results in shoot, compared to root, formation.

In a recent study, the action of cytokinins was evaluated in transgenic *Solanum* plants (which includes tomato, potato, and eggplant) that overexpressed isopentenyltransferase (IPT) in roots [15]. The enzyme IPT participates in the biosynthesis pathway of CKs. The results of this study showed that overexpressing IPT in tomato plants increased CK levels in leaves, in addition to delaying defoliation and increasing the plant chlorophyll concentrations after 18 days. The authors suggested that these effects are the action of CKs that act over long distances through shoot–root communication, using the xylem as a signaling conduit [15].

On the other hand, Palberg and colleagues [16] evaluated the production of CKs in 46 strains of bacteria of the genus *Methylobacterium*. Surprisingly, most of these strains produced high levels of CKs, including the most active form, trans-Zeatin (tZ). The authors also reported that the presence of carbon, derived from reduced methanol, in the bacterial medium was the only way to stimulate cytokinin production, and the strains were also able to produce indoleacetic acid (IAA) from L-tryptophan. In the future, it would be important to ascertain the effect that strains with high levels of production of cytokinins have on the bacterial interaction with plants, since various bacterial methylotrophs have been widely recognized as growth promoting agents, including under stressful conditions, in rice, sugar cane, mustard, potato, radish, peach, and groundnut [17].

Other genera such as *Pseudomonas* have also been reported as producers of CKs [18]. In this work, the authors found that the cytokinin-producing strain *Pseudomonas fluorescens* G20–18 enhanced tomato plant growth and boosted its tolerance to drought stress when the bacterium was used to inoculate the plant roots. Some plant parameters that increased in inoculated plants (compared to noninoculated plants) were a higher content of chlorophyll and abscisic acid (ABA) in the leaves, as well as greater stomatal closure. The activity of different enzymes of carbohydrate metabolism was also increased, and there was a significant increase in the activity of several different antioxidant enzymes. This was correlated with higher levels of secondary metabolites (e.g., anthocyanins, phenols, and flavonoids). Importantly, when two mutant strains of G20–18 that were defective in the synthesis of cytokinins (*CNT1* and *CNT2*) were used to treat plants, some plant parameters did not show any obvious differences compared to those of plants treated with the wild-type strain. However, these treated plants (especially in the case of *CNT1*) showed a decreased regulation in genes that respond to plant drought stress. Overall, this study concluded that bacterially produced CKs can contribute to the robustness of crops, as well as improve their resilience under stress conditions.

## 3. Gibberellin

**Figure 2:**
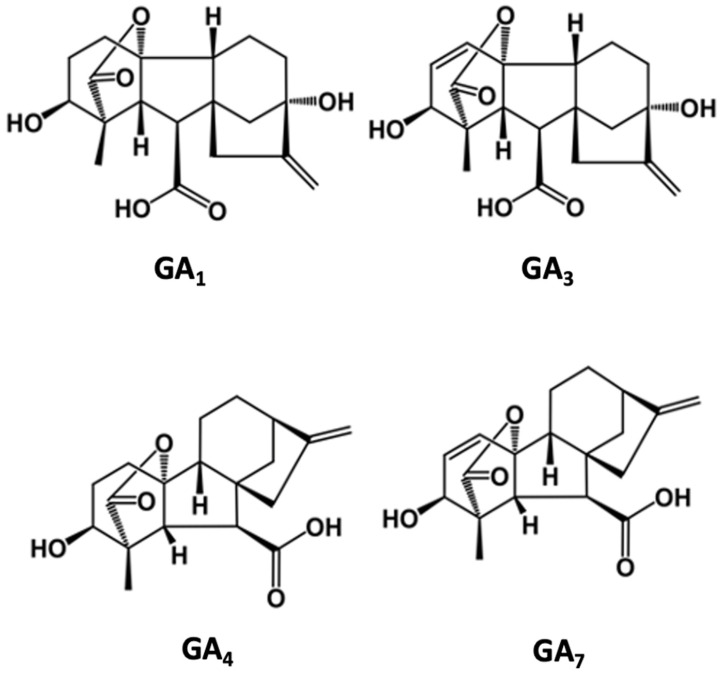
Chemical structures of bioactive gibberellins.

Gibberellins (tetracyclic di-terpenoid compounds) are a very large family of related compounds (there are ~126 known gibberellins). However, only four members of this family appear to have biological activity. The biologically active gibberellins are GA1, GA3, GA4, and GA7 (Figure 2). Gibberellin GA3 is the most common form of this phytohormone, and purified versions of this molecule are used commercially. The other (non-active) gibberellins may be involved in the biosynthesis or degradation of the active gibberellins [19].

Both purified and PGPB-synthesized gibberellins can increase plant stem growth, alter the dormancy of germinating seeds, and increase leaf and fruit senescence [19,20,21]. While the major effect of gibberellin appears to be the promotion of shoot growth, lower concentrations of gibberellin can also promote root growth [22]. Gibberellin (GA) production in beneficial (and pathogenic) plant-associated bacteria, such as PGPB, depends on the GA operon containing core genes *cyp112*, *cyp114*, *cyp117* (cytochrome P450 (CYP) monooxygenases), fd (ferredoxin), *sdr* (short-chain dehydrogenase/reductase), *ggps* (geranylgeranyl diphosphate synthase), *cps* and *ks* (two diterpene synthases/cyclases). The GA operon has been analyzed in the group of alphaproteobacterial rhizobia, which also can fix nitrogen, so both mechanisms are relevant in the interaction with host leguminous plants [23]. In fact, it has been suggested that there is a convergence of the genetic mechanisms involved in GA synthesis not only in bacteria, but also in plants and fungi. For example, fungi of the genera *Aspergillus*, *Cladosporium*, *Neurospora*, *Fusarium*, *Penicillium*, *Phoma*, among others, are GA producers under the control of genes with similar functions [19].

There are generally two types of GA: one group of molecules with 20 carbon atoms and another group of GA that includes a lactone ring and has 19 carbon atoms [24]. Importantly, as mentioned above, only four GAs have biological activity [25]. In this sense, Nett et al. [26] demonstrated that a *Bradyrhizobium diazoefficiens* GA three-oxidase enzyme is expressed in soybean (*Glycine max*) nodules, which participates in the last step in the synthesis of the bioactive GA_4_ by hydroxylating a non-active GA_9_, and was associated with an increase in the size of the nodules. A knockout mutant in the GA operon showed fewer large nodules and an increase in the number of small nodules. These authors suggested that the regulation of nodule size caused by the production of an active GA_4_ may be part of the co-evolution between the metabolism of the plant and its bacterial host where both can benefit from nodule formation.

As GAs are plant growth regulators, their own regulation must be finely orchestrated under different types of environmental stress. For example, in maize plants, the distribution of GA is determined by post-transcriptional modifications which increases the activity of the maize GA 20 oxidase (an enzyme involved in the biosynthesis of active GA) either in drought or cold stress conditions [27]. Since GA controls growth by regulating division–zone size and stresses like drought and cold alter leaf growth, this work reveals a greater understanding of GA metabolism and how factors such stressors could inhibit plant growth.

External application of purified GA can regulate and improve plant growth under abiotic stress. For example, Guo and colleagues observed that tomato (*Solanum lycopersicum* L.) plants sprayed with different concentrations of GA (50 and 75 mg L^−1^ of GA_3_) improved the shoot and root biomass, as well as increased the levels of proline, nitrogen, potassium, and phosphorous in its leaves [28]. It should be noted that the authors suggest that foliar application of GA at concentrations greater than 75 mg L^−1^ may not be optimal and have undesirable effects, so they recommend 75 mg L^−1^ as an optimal concentration to reduce heat stress and improve various physiological and performance characteristics like biomass increment in tomato plants.

**Figure 3:**
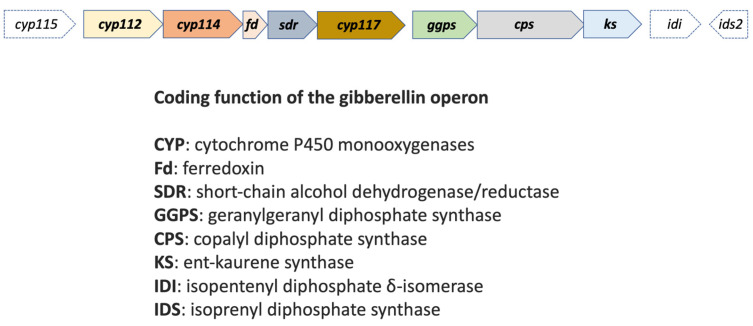
Coding functions of the gibberellin operon. Figure adapted from [29,30].

Figure 3 shows the coding functions of the GA operon genes, including the *cyp112*, *cyp114, fd, sdr, cyp117, ggps, cps,* and *ks genes*, which are almost always present in N-fixing rhizobia and other bacteria. Other genes, such as *cyp115*, *idi*, and *ids2*, have a more limited distribution within the bacterial genomes, including some pathogens like *Erwinia tracheiphila* or *Xanthomonas oryzae* [29].

## 4. Volatile Organic Compounds

While they are not considered to be phytohormones per se, the volatile organic compounds (VOCs) that are synthesized by a wide range of soil bacteria have recently been shown to play a significant role in plant growth promotion [6,31,32,33,34]. The growth promotion effect of VOCs is largely attributed to the modulation of the synthesis and/or metabolism of known phytohormones, synthesized by either PGPB or by plants [35,36]. The bacterial synthesis of VOCs has been shown to increase plant photosynthesis, and the content of gibberellin, auxin, and cytokinin, depending upon the plant and the particular organic compounds involved. Moreover, VOCs have been reported to decrease plant ethylene levels and to inhibit the functioning of several fungal phytopathogens (which in part function by increasing plant ethylene levels). By the year 2014, ~350 different soil bacterial species had been shown to produce nearly 900 different VOCs, and many more VOC-producing bacterial species having been reported since that time [37]. Importantly, VOCs have been found to positively affect the growth of a number of different plants.

For example, the volatiles produced by *Pseudomonas pseudoalcaligenes*, including dimethyl disulfide, 2,3-butanediol, and 2-pentylfuran, increased the growth of maize plants and alleviated stress symptoms caused by drought [38]. Another bacterium that also benefited the growth of maize (e.g., shoot and root dry weight) through the production of its volatile compounds was the Ab-V5 strain of *Azospirillum brasilense*. When Ab-V5 was co-inoculated with a strain of *Bacillus thuringiensis*, no synergistic or additive effects on plant growth were reported, compared to the controls.

Sorghum is another of the largest crop grains that is cultivated around the world, and it has also been reported that PGPB and their volatiles can increase sorghum growth directly or indirectly, through the inhibition of potential pathogens. In fact, Sudha et al. [39] reported the production of antifungal volatiles produced by *Streptomyces rochei*. Some of the VOCs detected had possible inhibitory action against sorghum grain mold pathogens such as *Fusarium moniliforme* and *Curvularia lunata* (with reductions of up to 63 and 68% of their mycelial growth), included 2-methyl-furan, benzene, 2-methyl-1-butanol, and myrcene. The authors concluded that *S. rochei* exhibits activities like hyperparasitism, competition, and antibiosis as a direct consequence of the VOCs that it produces.

When other volatiles were analyzed in four beneficial strains (*Pseudomonas koreensis* N19, *P. fluorescens* N04, *Lysinibacillus sphaericus* T19, and *Paenibacillus alvei* T22) the production of multiple molecules such as aldehydes, alcohols, ketones, alkenes, alkanes, acids, amines, pyrazines, furans, sulfides, terpenoids, and salicylic acid were observed. Some of these VOCs were species- and strain-specific. However, their effect on the plant was not evaluated, although some volatiles have previously been reported as plant growth stimulants [40,41].

The volatiles produced by PGPB are diverse in their structure, function, and plant growth stimulation mechanisms, and they modulate the expression of genes related to plant growth and development. Such is the case presented recently by He and colleagues [42]; they observed that a strain of *Streptomyces* sp. TOR3209 induced the expression of genes encoding protein molecules such as RING/U-box protein with C6HC-type zinc finger, UDP-glycosyltransferase (UGT), glutamate receptor, and leucine-rich repeat receptor-like protein kinase. These UGT genes are involved in mitotic processes in plant meristematic cells, while U-box proteins play important functions, such as in cell cycle regulation, morphogenesis, and regulation of the plant’s innate immune response when subjected to biotic stress. According to the authors, the expression of these genes was mainly due to the volatiles produced by the strain TOR3209, including two with antifungal action (i.e., 2,4-bis(1,1-dimethylethyl)-phenol and hexanedioic acid dibutyl ester) [42].

A recent study of the beneficial action of bacterial VOCs was reported by Venneman et al. [43] who found that endophytic strains of *Serendipita* spp. that produce VOCs are capable of improving the yield and biomass of *Arabidopsis* seedlings in in vitro experiments. Some *Arabidopsis* parameters that benefited from the mixture of VOCs that were emitted by the bacteria (and not by single VOCs) included petiole elongation, extension of the lateral root system epidermal cell and leaf area expansion, enhanced maximum quantum efficiency of photosystem II (Fv/Fm), and a high level of anthocyanin accumulation. Interestingly, it was found that the auxin and cytokinin signaling pathways may participate in the modulation of plant growth via the production of mixtures of volatiles produced by these endophytic strains.

It has been proposed that there are volatiles whose action is unique in stimulating certain plant tissues or organs; these include 2,3-butanediol or acetoin, which stimulate shoot biomass [44], while others such as dimethylhexadecylamine have antifungal effects [45,46]. However, most of these analyses have been carried out in vitro, so that it is necessary to evaluate the role of bacterial VOCs under real environmental conditions.

## 5. Salicylic Acid

Plants treated with PGPB often acquire broad-spectrum and long-lasting systemic resistance to a wide range of phytopathogenic fungi and bacteria [47,48,49,50]. This induced systemic resistance (ISR) primes plant defenses ahead of their interaction with phytopathogens so that plants are more resistant to subsequent pathogen attack. The ISR is often associated with an increase in the lignification of plant cells, as well as increases in the expression of enzymes that lower the level of reactive oxygen species (ROS) such as peroxidase, catalase, and superoxide dismutase. The induction of ISR is typically associated with ethylene and jasmonic acid signaling. In addition, phytopathogens themselves can often induce a defense response against pathogens in plants that is called systemic acquired resistance (SAR) where a group of plant pathogenesis-related (PR) genes that encode proteins with antipathogen activity (such as antibiotics and fungal cell wall degrading enzymes) are induced [51]. The induction of SAR is generally associated with salicylic acid (SA) signaling.

In addition to the involvement of salicylic acid in SAR, this hormone has been found to be involved in the mitigation of various plant abiotic stresses including both high and low temperature, high levels of salt, inhibition by high levels of metals, insufficient levels of oxygen, ozone, the presence of toxic organic chemicals, ultraviolet radiation, and drought [52]. For example, Aires and colleagues [53] applied SA to tomato leaves subjected to water stress and observed a favorable plant response by improving parameters such as CO_2_ assimilation, transpiration, stomatal conductance, water use efficiency, and carboxylation efficiency. Two of the above positive effects, gas exchange and the efficiency of CO_2_ assimilation and carboxylation, were reflected in an improvement in tomato fruit production. The tomato fruit production was followed for two years of good results, demonstrating a fundamental role of SA in this process. Similar results were reported in wheat plants (*Triticum aestivum* L.) grown under hydroponic conditions. The SA was applied exogenously to plants subjected to water stress, resulting in a positive impact on plant growth and the induction of stress tolerance [54].

Salicylic acid can stimulate flowering, ion absorption, nutrient transfer, the movement of plant stomata, and protein synthesis [55]. Salicylic acid can bind to certain amino acids such as proline and arginine and as a result increase a plant’s ability to resist several environmental stresses. Given its role in both SAR and mitigation of abiotic stress, salicylic acid may be described as a defense hormone. In one study, the addition of salicylic acid, along with PGPB, to chickpea plants under high salt stress significantly increased the ameliorative response of the PGPB by itself [56]. In this and other studies, salicylic acid appeared to function synergistically with PGPB-encoded mechanisms [57,58]. Mehrasa et al. [59] co-inoculated two PGPB strains (*Bacillus subtilis* and *Pseudomonas putida*) and SA with white bean plants with the result that plant growth under drought stress conditions was improved in comparison to plants untreated with SA. In addition, other parameters such as relative water content, proline synthesis, nitrogen content, and chlorophyll content, were improved by the co-application of SA and the abovementioned endophytic bacteria.

A similar strategy of co-application of SA and a PGPB (*Bradyrhizobium* sp. strain W100) onto four cowpea genotypes was performed by de Andrade et al. [60]. These authors found positive impacts of SA addition on the growth in three plant genotypes that received this co-application when subjected to water stress. Some parameters such as leaf water potential and proline production, as well as other antioxidant activities were stimulated.

Other researchers have also reported positive effects on plant growth with the application of SA and other compounds, such as potassium silicate (silicon), melatonin (MT) or the disaccharide trehalose in plants subjected to different types of stress. In the first case, silicon and SA were observed to play an important role in tomato plants in their resistance to the pathogenic effects of the Gram negative bacterium *Ralstonia solanacearum* [61], while the co-application of MT and SA improved growth in wheat plants subjected to salt stress. The co-application of MT and SA markedly improved plant functions such as the photosynthetic pigments content, photochemical reactions of photosynthesis, net photosynthetic rate, transpiration rate, stomatal conductance, as well as the osmoprotectants accumulation [62]. In the case of trehalose, it is well known that this disaccharide, which is produced by various species of PGPB, plays important roles in plant and microbial resistance to heat, drought, salt, cold stress, or even resistance to biotic factors, such as bacterial pathogens [63,64,65]. In this case, the application of trehalose and SA decreased the negative effects of drought stress on sweet basil plants. The plants responded positively by improving their production of osmolytes (glycine and glycine betaine), some antioxidant enzymes (superoxide dismutase, peroxidase, and catalase), as well as their general growth under drought stress conditions [66].

## 6. Auxin

Historically, PGPB synthesized auxin is considered to be the major mechanism that the bacteria use to facilitate plant growth. Moreover, among several auxins with biological activity, most of the scientific literature is concerned with indoleacetic acid (IAA) so that frequently the terms IAA and auxin are used interchangeably. IAA promotes a wide range of plant growth traits, including both root and shoot growth, cell expansion, root bacterial colonization, differentiation of vascular tissues, defense against pathogens, stimulation of cell division, elongation of stems and roots and loosening of root cell walls [67,68,69,70,71,72,73,74]. Different plant tissues respond optimally to different IAA concentrations that is needed for maximal stimulation of shoots being around 100,000 times greater than the concentration required for maximal root growth. The IAA is often found in plants in a conjugated (and inactive) form with this conjugated form typically comprising ~75% of the total IAA within a plant.

Importantly, a very large percentage (~80–90%) of characterized PGPB have been found to synthesize IAA suggesting that IAA is a key component of the mechanism(s) used by these bacteria to promote plant growth and development. Moreover, based on a combination of biochemical and genetic studies, there are at least five separate metabolic pathways for the synthesis of IAA that are found in various bacterial strains [72,73,75]. In addition, numerous characterized PGPB have been shown to contain several IAA biosynthetic pathways and these pathways often intersect (overlap) with one another [76]. This presumably reflects the importance of IAA to the functioning (i.e., promoting plant growth) of these PGPB. The rationale for the presence of multiple IAA biosynthetic pathways may be the fact that deleterious mutations in one pathway will not prevent the functioning of the other biosynthetic pathway(s) and will therefore not decrease the effectiveness of the PGPB strain in facilitating plant growth and development. This is relevant in agriculture, since the application of biostimulants based on auxin producing PGPB, such as IAA, typically have beneficial effects on plant growth and development. In fact, the production of IAA has been reported in various genera of PGPB including *Acetobacter*, *Acinetobacter, Azospirillum, Arthrobacter, Azotobacter, Bacillus, Bradyrhizobium, Burkholderia, Herbaspirillum, Klebsiella, Mesorhizobium, Paenibacillus, Pantoea, Pseudomonas, Rhizobium, Rhodococcus, Serratia, Strenotophomonas, Streptomyces,* and *Rouxiella* [77,78].

Another important function of the production of IAA in PGPB is the improvement of the solubilizing capacities of elements such as phosphate, which is another mechanism that improves the uptake of inorganic phosphate by plants, particularly in soils where there are solubilization problems [79]. It is associated with beneficial actions in barley and chickpea plants of IAA production in three diazotrophic strains of the genus *Klebsiella* and their nitrogen utilization efficiency (NUE) [63]. This conclusion was derived mainly from the comparison between a non-IAA-producing strain and two that did produce IAA, observing that the IAA-producing strains stimulated root growth in both plant species much better than the strain lacking IAA synthesis.

As previously mentioned, IAA is one of the most common and studied hormones in various plant crops, which usually include those producing vegetables, fruits, and grains [80]. However, IAA also has relevant functions in the regulation of growth and development in aromatic, medicinal, and woody plants [81,82]. In the latter case, the auxin-line metabolite-producing bacterium, *Pantoea agglomerans*, enhanced root emergence and improved root architecture in two woody species, including *Pyrus communis* L (pear) and hazelnut, when inoculated into these seedlings. In addition, some auxin response genes were overexpressed, including *PcARF6*, *PcARF7*, and *PcARF8*, compared to actin genes that were used as reference controls [82].

## 7. Ethylene

Ethylene is a ubiquitous gaseous phytohormone with a wide range of biological activities that function over an even wider range (~10,000-fold) of concentrations. Higher ethylene levels are typically found in ripening fruit while much lower levels are associated with early vegetative development, seed germination, tissue differentiation, formation of root and shoot primordia, root branching and elongation, lateral bud development, pollen tube growth, flowering initiation, anthocyanin synthesis, flower opening and senescence, VOC synthesis, leaf and fruit senescence, Rhizobia nodule formation, mycorrhizae–plant interaction, and the response of plants to various biotic and abiotic stresses [83,84,85]. It was recently postulated that ethylene regulates growth responses using different signaling pathways, such as auxins and abscisic acid (ABA), whose responses can reduce the ability of roots to penetrate the soil, mainly in compacted soils [86].

Ethylene biosynthesis in plants begins with the compound *S*-adenosylmethionine (SAM) being converted to 1-aminocyclopropane-1-carboxylic acid (ACC) by the enzyme ACC synthase followed by ACC being converted to ethylene by the enzyme ACC oxidase [83]. During periods of time when there is an excessive amount of ACC within plant tissues, this compound typically becomes conjugated and converted into inactive forms. This is especially important since some recent evidence indicates that ACC per se (in addition to ethylene) may possess some signaling activity that was previously attributed to ethylene [84,87].

Many PGPB contain the enzyme ACC deaminase which is capable of degrading ACC to ammonia and alpha-ketobutyrate [88,89]. A model that explains the functioning of ACC deaminase in plant growth promotion includes IAA from a PGPB being taken up by a plant and (together with the endogenous plant IAA) promoting growth and, at the same time, facilitating the transcription of plant ACC synthase resulting in an increased level of ethylene thereby inhibiting plant growth [90,91,92]. Much of the increased ACC following IAA stimulation of ACC synthase transcription is taken up by the ACC deaminase containing PGPB and is subsequently cleaved and deactivated. Thus, because of the action of the PGPB, ACC levels and hence plant ethylene levels are lowered, and IAA can continue to promote plant growth without producing an inhibitory increase in ACC.

Given its critical role in regulating plant ethylene levels especially during periods of both biotic and abiotic stress, the functioning of ACC deaminase is one of the key mechanisms that PGPB use to facilitate plant growth and development [93]. This is demonstrated by the work of Araya et al. [94] where they isolated and characterized bacterial strains from the rhizosphere, endosphere, and phyllosphere of plants that inhabit Antarctica. In this study, 77 strains were both cold tolerant and ACC deaminase producers. Some isolates were categorized as “cold-tolerant and hyper-ACC-degrading bacteria”, which included genera such as *Pseudomonas*, *Serratia*, and *Staphylococcus*, the most investigated as bioinoculants in cold-stressful agriculture.

Plants are subject to various types of environmental stress, some of which have already been mentioned, such as drought and soil salinity. However, the cultivation of plants such as rice, which are typically grown under waterlogged soils, can also be a form of stress where ethylene levels may be increased. For this reason, the application of ACC deaminase-producing bacteria, such as *Paenibacillus* sp. ANR-ACC3 and *Methylophaga* sp. AR-ACC3, are efficient in relieving such stress in rice plants due to submergence, even showing growth stimulation in parameters such as seedling vigor, root and shoot length, and total chlorophyll concentration [95].

PGPB contain various mechanisms for promoting plant growth, but few studies have tried to understand the connections that exist between them. For example, Alemneh et al. [96] investigated the connection between ACC deaminase production and phosphate solubilization in PGPB, including *Bacillus*, *Burkholderia*, *Pseudomonas*, and *Variovorax*. Plant experiments showed that *Burkholderia* sp. strain 12F, expressing ACC deaminase, improved chickpea nodulation and growth by also stimulating phosphorus uptake from rock phosphate.

The ACC, the precursor of ethylene, is exuded from plant tissues during stress, including roots in the rhizosphere [97], and can then act as a chemoattractant for rhizobacteria [98], among other metabolites produced by the plant. However, as ACC deaminase is present in the microbial cell cytoplasm [88], ACC can be transported by exopolysaccharides, chelators, peptides/chaperones, hormones, or concentration gradient differences into the ACC deaminase-containing microbes. This last mechanism would maintain the balance between the ACC present in the plant tissues, the rhizosphere ecosystem, and the ACC deaminase-producing bacteria. In addition, this attraction and transport mechanism could be important for other ACC deaminase-producing microorganisms, including fungi and archaea [99].

One study analyzed the overexpression of *acdS* genes in the PGPB *Pseudomonas* sp. UW4 which increased the bacterial ability to colonize the rhizosphere and stimulate the growth of wheat plants. The authors of that study concluded that ACC which was produced and excreted by the plant can be a very powerful chemoattractant for microbial strains with high levels of ACC deaminase activity [100]. This study is consistent with the work of Moon and Ali [99] mentioned above as well as a model of ACC deaminase functioning postulated earlier [90].

Other systems such as quorum sensing (QS) may also be important for regulating cell numbers in the rhizosphere of ACC-producing bacteria. This is demonstrated by the work of Jung et al. [101] in which the bacterium *Serratia fonticola* GS2 and QS-deletion mutants of this strain were constructed and tested. These strains revealed that QS functions can influence the growth promoting activities of PGPB. Some of the mechanisms affected in the QS mutants include the production of ACC deaminase, biofilm production, and IAA synthesis.

The search and screening of ACC deaminase producing PGPB is a fundamental part of finding the best candidates to be part of bioinoculants, which, due to the multiple functions exhibited by ACC deaminase activity, can be called biostimulants, biofertilizers or biofungicides. For this reason, detecting genes (e.g., *acdS*) and ACC deaminase functions in PGPB has been a subject of numerous studies and reports on the literature. Pioneering works such as that of Duan et al. [102] investigated the presence of *acdS* genes and their ACC deaminase activity in more than 200 strains isolated from the Canadian Prairies. Techniques such as PCR amplification, Southern hybridization and phylogenetic analysis revealed the existence of these genetic elements in various previously uncharacterized strains, most of which were nitrogen-fixing *Rhizobia*.

More recently, Bouffaud et al. [103] carried out an extensive screening of *acdS* genes in ACC deaminase-producing strains associated with maize, among other plant species (mainly the Poaceae family). For this purpose, the authors designed specific primers for the amplification of *acdS* genes, as well as quantification of the presence of mRNA encoding this enzyme in different soil communities in *acdS*^+^ microorganisms of the same plant species. The results showed that *acdS* genes and gene transcripts in Poaceae rhizospheres were commonly present. These results show that these genes are typically related to the capacity that rhizosphere bacteria must promote plant growth, as well as the potential for survival in such agroecosystems. A beneficial evolutionary relationship could exist between these Poaceae species and their rhizosphere microorganisms exhibiting ACC deaminase activity.

## 8. Conclusions and Perspectives

Plants have developed different mechanisms to respond to environmental stress, both abiotic (e.g., salinity, drought, submergence, temperature) and biotic (i.e., pathogen infection), using phytohormones. However, they also contain systems that respond to a great molecular diversity of phytohormones. Such is the case of Skp1/Cullin/F-box-type ubiquitin ligase that detects and responds to jasmonic acid, auxins (IAA), gibberellic acid, and strigolactones. Therefore, some authors have proposed more research on aspects of these interactions that are not only functional, but also have an evolutionary relevance [104]. This could reveal new similarities (and differences) of signaling the great diversity and functionality of phytohormones, which are also produced by a plant-associated microbiome, including PGPB.

It is evident that, based on all current evidence, the phytohormones produced by PGPB play an essential role in helping plants to tolerate certain stressful environmental factors [3]. However, one should ask how bacteria have co-evolved their hormone production and modulation systems, and how plants influence and regulate the synthesis of these bacterial hormones under different environmental conditions. This could be studied through -omic responses in both PGPB and in their associated plants [105]. In fact, it has recently been suggested that it is possible to infer causal relationships between plant microbiota, including plant growth modulating bacteria, and the desired phenotypes of plant crops [106].

Finally, the plant–PGPB relationship still has many aspects that require more detailed investigation; but it must be considered that this basic research is an essential precursor to the more widespread practical application of this technology.

## Data Availability

Not applicable.
